# Supplementary material for: Bio-Organic Fertilizer Application Enhances Silage Maize Yield by Regulating Soil Physicochemical and Microbial Properties
Source: Microorganisms. 2025 Apr 23;13(5):959. doi: 10.3390/microorganisms13050959 (PMC12114197; doi:10.3390/microorganisms13050959)
Supplement: Supplementary file 1 [file microorganisms-13-00959-s001.zip › microorganisms-3556558-supplementary.pdf]

Table S1. Irrigation and Fertilizer Application Plans for Silage Corn by Crop

Irrigation			Fertilization	
Irrigation Times	Corresponding Period	Irrigation Amount (m^3 /time)	N 382.5kg/ha + K_2O 105 kg/ha	
			Urea (kg/ha)	Potassium Chloride (kg/ha)
1st Time	Around April 20, Planting	40	52.5	0.7
2nd Time	Mid-May, 5-leaf Stage of Corn	20	0	0
3rd Time	Late May to Early June, Jointing Stage of Corn	30	7	2
4th Time	Mid-June	30	6	3.5
5th Time	Late June, Tasseling Stage of Corn	30	11	2
6th Time	Early July	30	10	1.5
7th Time	Mid-July	40	7	1.5
8th Time	Late July	30	6	1.4
9th Time	Early August	30	5	1.4
10th Time	Mid-August	30	0	0
Total:		310	55.5	14

Note: Irrigation is approximately once every 7 days. Fertilization is done 9 times, with corn fertilization according to the following schedule: Planting Start Fertilizer (5%, 1st Irrigation), Jointing Stage (20%, 3rd and 4th Irrigation), Tasseling Stage (35%, 5th and 6th Irrigation), Silk Earing Stage (20%, 7th and 8th Irrigation), Grain Filling Stage (20%, 9th and 10th Irrigation).

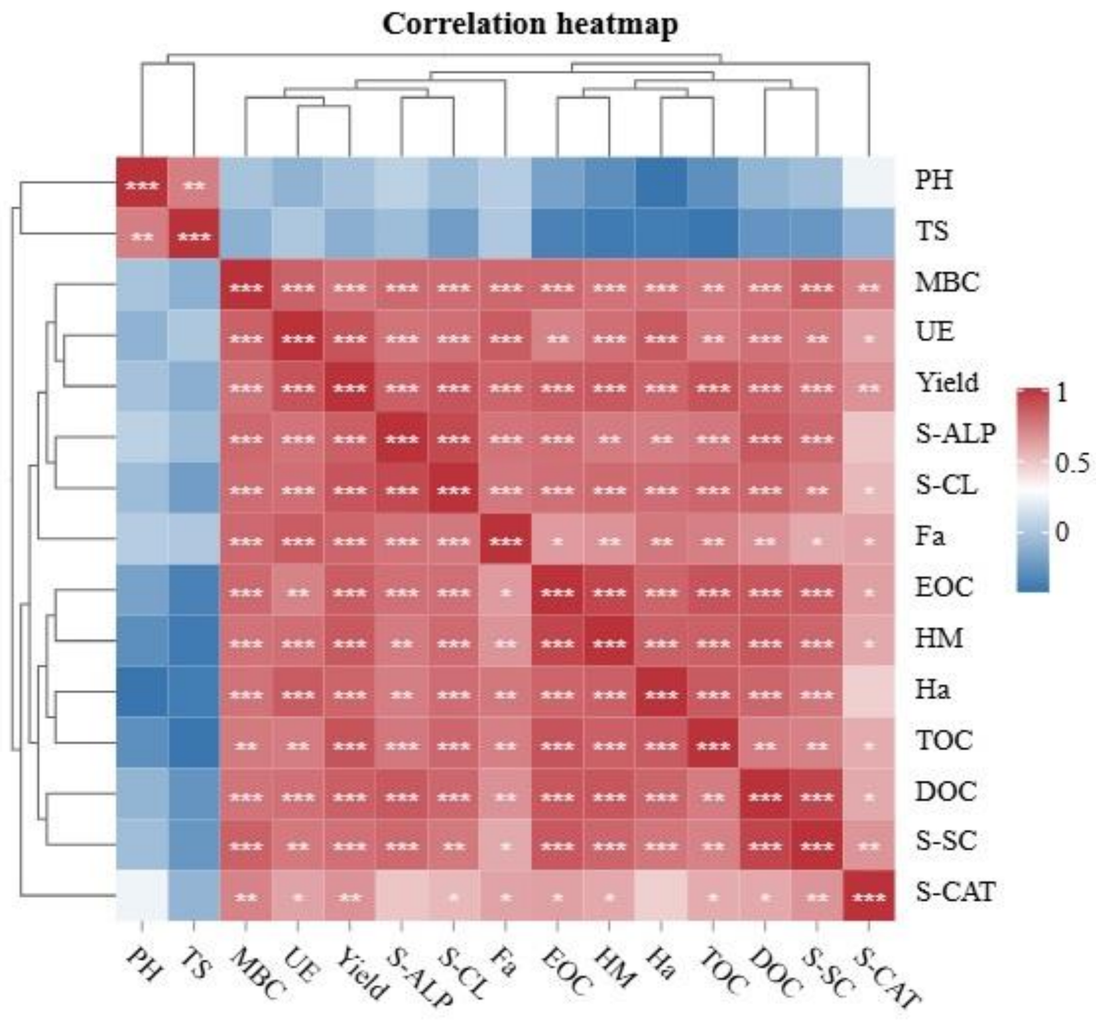

Figure S1. Correlation analysis between silage corn yield and soil physical and chemical factors
